# New focuses on roles of communications between endoplasmic reticulum and mitochondria in identification of biomarkers and targets

**DOI:** 10.1002/ctm2.626

**Published:** 2021-11-02

**Authors:** Linlin Zhang, Furong Yan, Liyang Li, Huirong Fu, Dongli Song, Duojiao Wu, Xiangdong Wang

**Affiliations:** ^1^ Zhongshan Hospital Department of Pulmonary and Critical Care Medicine Jinshan Hospital Centre for Tumor Diagnosis and Therapy Fudan University Shanghai Medical College Shanghai Institute of Clinical Bioinformatics Shanghai Engineering Research for AI Technology for Cardiopulmonary Diseases Shanghai China

**Keywords:** communications, contacts, diseases, endoplasmic reticulum, homeostasis, mitochondria

## Abstract

The communication between endoplasmic reticulum (ER) and mitochondria (Mt) plays important roles in maintenance of intra‐ and extra‐cellular microenvironment, metabolisms, signaling activities and cell‐cell communication. The present review aims to overview the advanced understanding about roles of ER‐Mt structural contacts, molecular interactions and chemical exchanges, signal transmissions and inter‐organelle regulations in ER‐Mt communication. We address how the ER‐Mt communication contributes to the regulation of lipid, amino acid and glucose metabolisms by enzymes, transporters and regulators in the process of biosynthesis. We specially emphasize the importance of deep understanding about molecular mechanisms of ER‐Mt communication for identification and development of biology‐specific, disease‐specific and metabolism‐specific biomarkers and therapeutic targets for human diseases. The inhibitors and modulators of the ER‐Mt communication are categorized according to therapeutic targets. Rapid development of biotechnologies will provide new insights for spatiotemporally understanding the molecular mechanisms of ER‐Mt communication.

## Graphical abstract

Highlights

1. The ER‐Mt interactions are the important part of intracellular communication responsible for cell metabolism, development and death in the biological processes.

2. The ER‐Mt regulations contribute to molecular mechanisms of diseases and vary among diseases, severities, stages and sensitivities to therapies.

3. The molecular signals during ER‐Mt communication can be potential sources to discover and develop biology‐, disease‐ and therapy‐specific biomarkers and targets.

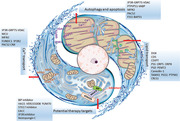

## 1 INTRODUCTION

The multiple functions of endoplasmic reticulum (ER), as the largest membrane‐bound organelle in eukaryotic cells, have been studied for decades. The different domains between ERs and other organelles are the membrane contact sites. Of those, the ER‐ mitochondria (Mt) contact site is directly or indirectly responsible for the communication between cells. Single‐cell RNA sequencing provides important information on ligand‐receptor interactions and networks for understanding, cell‐cell communication in the homeostasis of the microenvironment.^1^ For example, the cell‐cell communication can be featured by increasing synthesis of lipoproteins and ceramides and transport of lipids between ER and Mt through Mt‐associated ER membranes (MAMs) and multivesicular endosomes. The activation of MAMs and endosomes promote the synthesis of ceramide, Adenosine Triphosphate (ATP) flux between ER and Mt and secretion of exosomes.^2^ Exosomes and lipoproteins are important mediators of cell‐cell communications.^2^ Of those, the contact and communication between ER and Mt as the most important energy producer play decisive roles in cell metabolism, biosynthesis and function. The ER ‐Mt contact was first described by electron micrographs in 1950s,^3^, ^4^ and molecular structures and functions of such contact were defined about 40 years later.^5^ The density of the communications between ER and Mt membranes and connecting molecules varies among species.^6^ The ER‐Mt communication includes ER‐Mt structural contacts, molecular interactions, chemical exchanges, signal transmissions and inter‐organelle regulations. In addition, ER also has contact sites with the Golgi apparatus, peroxisomes and nuclear membrane.^7^, ^8^ The current review aims to recall the special attentions to understanding of molecular mechanisms by which ER‐Mt communication regulates and controls cell metabolism, lipid biosynthesis and structure and function of ER‐Mt contact sites. We also address the importance of transporter and regulator contributions to ER‐Mt communication and discover of potential therapeutic and biological targets.

## 2 ER‐MT COMMUNICATION AND HETEROGENEITY

The Mt‐associated ER membrane (MAM) is one of the multiple membranes contact sites in yeast and mammalian cells. The MAM function includes the exchange of lipids, ions, and second messengers, mitochondrial fission and induction of autophagy. For yeast and mammals, the contact between ER‐Mt has unique functional significance. Although the functions of MAM in yeast *S. cerevisiae* and mammalian cells partially overlap, there are important differences between these two species.^4^ The ER‐mitochondrial encounter structure (ERMES) as a multi‐protein complex contains the mitochondrial distribution and morphology (Mdm)‐10, 12, 34 and ER‐anchored maintenance of mitochondrial morphology 1 (Mmm1) proteins, responsible for ER‐Mt communication in yeast.^9^ The protein‐protein interaction among core components creates a binding force between ER and Mt to maintain a close distance. Some ERMES complex binding proteins are identified as auxiliary subunits of the ERMES complex, such as Ca^2+^ binding to GTPase Gem1 and Mdm10 binding to outer membrane 7 translocase. Gem1 is necessary for the formation of MERCS, although the function of translocase in regulating MERCSs remains unclear.^10^ During the connection, soluble lipid carrier proteins such as ceramide transfer protein and oxysterol binding protein carry out lipid transport in mammalian cells.^11^ The ER‐Mt connexin vesicle‐associated membrane protein‐associated protein B (VAPB) is an indispensable ER protein from yeast to human.^12^ The transport of phosphatidylserine from ER to Mt requires ATP in mammalian cells, rather than in yeast.^4^ In mouse hepatocytes, at least in one Mt of four have MAMs. However, in the Mt located at the synapses of neurons in the dentate gyrus of the mouse brain, MAM is usually absent.^13^ The tension of ER‐Mt communication (e.g., contact area, tightness, distance, length, number, localization and regulator activity) plays an important role in the maintenance of cell homeostasis.

## 3 ER‐MT COMMUNICATION WITH CONTACT SITE PROTEIN

**FIGURE 1:**
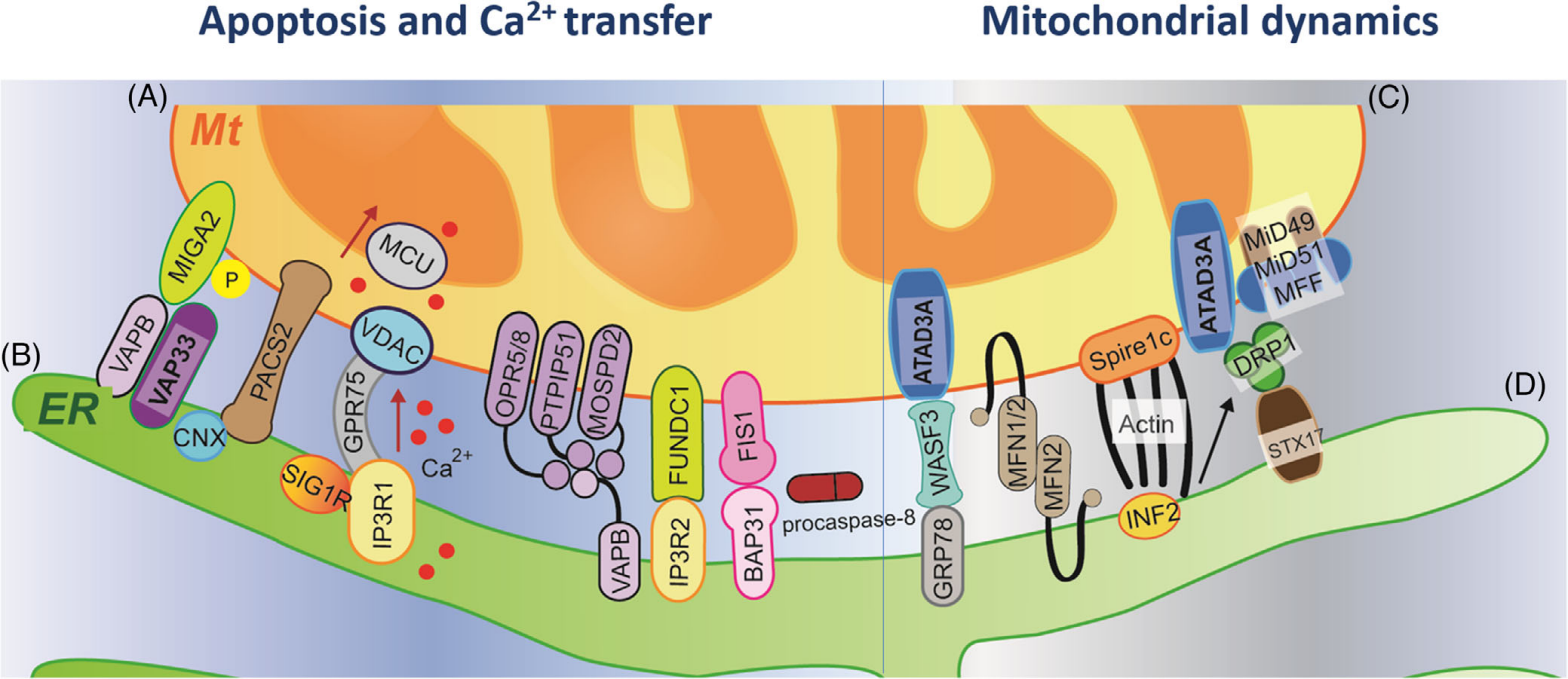
The main molecular domains located on the ER‐ Mt contacts sites were summarized. VAPB can bind to several mitochondrial proteins including MIGA2, OPR5/8, PTPIP15 and MOSPD2 (A). These tethers involve their role in the delivery of Ca^2+^ to mitochondria from ER. PACS2 is a cytosolic multifunctional sorting protein, essential for ER‐mitochondria Ca^2+^ transfer and apoptosis by the recruitment of CNX. GRP75 links IP3R1 on ER and VDAC1 on OMM to regulate Ca^2+^ efflux from ER to mitochodnria. FUNDC1, an integral mitochondrial outer‐membrane protein, can bind IP3R2 to modulate Ca^2+^ efflux from ER. FIS1 can convey an apoptosis signal from the mitochondria to the ER by interacting with BAP31, recruiting and activating procaspase‐8 (B). WASF3 can interact with ATAD3A and GRP78, which may provide a bridge between ER and mitochondria. MFN2 on the ER can bridge the two organelles by engaging in homotypic and heterotypic complexes with MFN1/2 on OMM (C). Mitochondrial fission occurs at ER‐mitochondria contact site where Spire1c promotes actin assembly on the surface of mitochondria through interacting with INF2 on ER. Then, DRP1 are recruited and interact with MFF and/or MiD94/51. ATAD3A dimerization is required for DRP1‐mediated mitochondrial fission. In addition, DRP1 can interact with STX17, playing an important role in mitophagy (D). Abbreviations: Mt, mitochondria; ER, endoplasmic reticulum; VAPB, vesicle‐associated membrane protein‐associated protein B; MIGA2, mitoguardin 2; OPR, oxo‐phytodienoic acid reductase; PTPIP15, protein tyrosine phosphatase‐interacting protein 51; MOSPD2, motile sperm domain‐containing protein 2; PACS2, phosphofurin acidic cluster sorting protein 2; CNX, calnexin; GRP, glucose‐regulated protein; VDAC, voltage‐dependent anion channel 1; OMM, outer membrane of mitochondria; FUNDC1, FUN14 domain containing 1; IP3R, inositol 1,4,5‐trisphosphate receptor; FIS1, fission protein 1; WASF, Wiskott–Aldridge syndrome family; ATAD, ATPase family AAA‐domain containing protein; MFN, mitofusin; INF, inverted formin 2; DRP1, dynamin‐related protein 1; MFF, mitochondrial fission factor; STX17, syntaxin‐17; MCU, mitochondrial calcium uniporter; BAP31, B cell receptor‐associated protein 31; Spire1C, splice‐isoform of Spire1

The protein is a core element for the formation of MAM structure, where approximately 1000‐2000 proteins are identified using proteomics, indicating a rich signaling platform with multiple functions between organelles.^14^ The main molecular domains on the ER‐Mt contact sites are summarized in Figure 1. The distance between ER and Mt is approximately 10–30 nm, and the ER‐Mt contact can be converted to less than 10 nm under the cellular pressure,^15^ although the mechanism remains unclear. On the ER‐Mt interface with four major players in mammalian cells, the voltage‐dependent anion channel 1 (VDAC), fission protein 1 (FIS1) and phosphatase interacting protein 51 (PTPIP51) on Mt membrane site (Figure 1A) coupled with inositol 1,4,5‐trisphosphate receptor (IP3R)‐ glucose‐regulated protein 75 (GRP75)‐ VDAC, B cell receptor‐associated protein 31 (BAP31), and mitofusin 2 (MFN2) and VAPB‐ phosphatase interacting protein 51 (PTPIP51) on ER membrane site (Figure 1B), respectively.^16^ The outer membrane of mitochondria (OMM) channel VDAC1 activates the Mt Ca^2+^ channel on the microdomain, increasing the influx of Ca^2+^ into the membrane space and Mt through the opening ER‐resident Ca^2+^ release channel IP3Rs.^17^ GRP75 in the cytoplasm is a modulator of the IP3R‐VDAC complex, to increase the efficiency of Mt Ca^2+^ uptake by promoting the interaction between channels.^18^ In Parkinson's disease, DJ‐1 interacts with IP3R3, GRP75 and VDAC1 on MAM and forms a macro‐complex at the ER‐Mt contact site. The loss of DJ‐1 destroys such macro‐complex and reduces the level of the IP3R3‐GRP75‐VDAC1 tripartite complex in vitro and in vivo.^19^ Such Ca^2+^ transferring between ER and Mt is responsible for the formation of apoptosis, while WASF3, MFN1/2, actin and DRP1 bridge Mt (Figure 1C) and ER (Figure 1D) for the function of Mt dynamics.

The mammalian ER membrane contact sites cover about 2%–5% of the Mt surface area, to perform the diversity functions between Mt and ER. The Mt are tightly tethered to the ER, a critical step for organelles to ‘drag' ER tubules during inter‐organelle communication, even if there is a distance between organelles, rather than fusing with ER. The dynamics and structure of ER are affected by the movement of organelles.^20^ With the development of the methodology to isolate MAMs,^21^ it is possible to deeply explore molecular mechanisms of regulator involvement, phase separation dynamics of sphingomyelin and phospholipids into a rigid membrane domain in ER, and formation of functional domains with an affinity for different categories of lipids.^22^, ^23^ Altered MAMs are responsible for the maintenance of Mt shape and motility as well as development of autophagy and apoptosis. The spatiotemporal biology of MAMs remains unclear due to the limitation of ER isolation, definition of target gene/protein‐specific networks and complexity of ER‐Mt communications, although ER‐Mt contact sites for ion and lipid transfer, signaling and membrane dynamics were identified for years.^24^, ^25^ The ER membrane protein complex and structure contain the membrane spanning region with cores, basket‐shaped cytosolic region and L‐shaped luminal region. Altered complex structure causes the instability and the insertion of post‐ and co‐translational complex‐dependent substrates, resulting in the hydrophilic vestibule disorder of ER membrane protein complex.^26^

## 4 ER‐MT COMMUNICATION WITH LIPID TRANSPORTS

**FIGURE 2:**
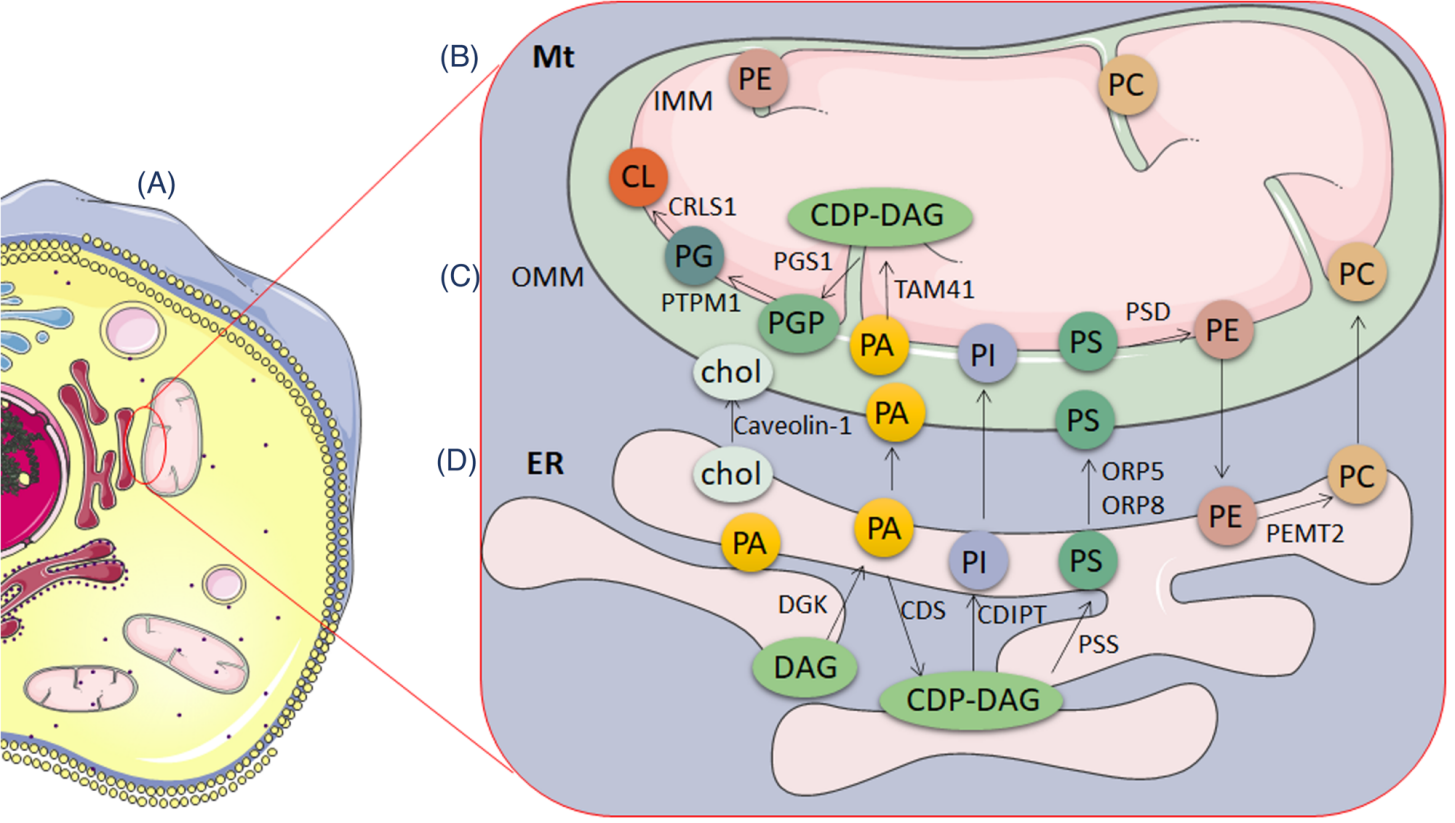
The synthesis and transport of lipids through the contact between Mt and ER also called MAM or Mt‐ER contact (MERCs) (A). The accumulation of cholesterol and sphingolipids in MAM is related to increased Caveolin‐1, which regulates the transfer of ER‐mitochondrial cholesterol. DAG is converted to PA by DGK. DAG is the predecessor of PI and PS in ER, to synthesize PI and PS through PA. PS is synthesized in the ER by the MAM enzymes PSS1 and PSS2. ER provides PI and PS to Mt, which are transferred through lipid transfer proteins ORP5 and ORP8 (B). After then, the newly formed PS is transferred to the inner Mt membrane through MAM. IMM contains PSD, which converts PS to PE. Therefore, PS is transferred to OMM first, transferred to IMM and then converted to PE. Subsequently, PE returns to the ER, where PEMT2 mediates the synthesis of PC, and the PC is transferred from the ER to Mt. PA is an important raw material for the synthesis of CL. It is transferred from ER to OMM, then to IMM and converted to CDP‐DAG through Mt TAM41 in IMM. CDP‐DAG is catalyzed by PGS1 to synthesize PGP, and further through PTPM1 to generate PG, which synthesizes CL under the catalysis of CL synthase CRLS1 (C and D). Abbreviations: IMM, inner mitochondrial membrane; OMM, outer mitochondrial membrane; PA, phosphatidic acid; PS, phosphatidylserine; PC, phosphatidylcholine; PI, phosphatidylinositol; Chol, cholesterol; PG, phosphatidylglycerol; PE, phosphatidylethanolamine; CL, cardiolipin; CDP‐DAG, cytidine diphosphate diacylglycerol; PGP, glyceraldehyde 3‐phosphate; PSD, PH and SEC7 domain‐containing protein 1; DGK, diacylglycerol kinase; PSS, phosphatidylserine synthase; ORP, OSBP‐related protein; TAM41, translocator assembly and maintenance protein 41 homolog; PEMT2, phosphatidyle thanolamine N‐methyltransferase 2; CRLS1, Cardiolipin Synthase 1; PGS1, Phosphatidylglycerophosphate Synthase 1; PTPM1, phosphatidylglycerophosphatase and protein‐tyrosine phosphatase 1; CDS, CDP‐diacylglycerol synthases; CDIPT, CDP‐diacylglycerol‐inositol 3‐phosphatidyltransferase

Biosynthetases coordinate major cellular phospholipid synthesis on the ER membrane and Mt matrix.^20^ The ER contacts with mitochondria for proper lipid synthesis and transport through the molecular activation on ER‐Mt contact sites (Figure 2A), including cholesterols, fatty acids and conjugates, and terpenoids. Of those, cholesterols are synthesized, up‐taken and esterified in ER and endosomes, while catabolized in Mt. The synthesis of phospholipids occurs in specific ER subdomains (Figure 2D) and contacts with Mt (Figure 2B) through OMM (Figure 2C) between two organelles.^5^ For example, phosphatidylserine is synthesized in ER, transported to mitochondria by lipid transporter Oxysterol‐binding protein (OSBP)‐related protein 5/8, and the metabolized by mitochondrial‐localized phospholipid synthase into phosphatidylethanolamine. Subsequently, the phosphatidylethanolamine produced in Mt is delivered to ER and converted into phosphatidylcholine by ER‐localized enzymes.^27^ The ER is the critical platform for the endogenous biosynthesis of cholesterol as a precursor for bile acids and steroid hormones, low‐density‐lipoprotein receptors for the transport of exogenous cholesterol into the cell and Niemann‐Pick Protein C1 and C2 for cholesterol from the lysosomal membrane to ER. Of participates in the process, acyl‐CoA: cholesterol acyltransferase 1 as an ER enzyme transfers a fatty acyl group from acyl‐CoA to the 3β‐hydroxyl moiety of cholesterol, forming cytoplasmic lipid droplets. The acyl‐coA:cholesterol acyltransferase 1 holoenzyme is a tetramer with two homodimers related to C2 symmetry, nine transmembrane helices, and one cavity with histidine for drug binding and cholesterol esterification.^28^ Different from cholesterol transport, the synaptotagmin‐like Mt‐lipid binding protein domain and two adjacent C2 domains at ER‐Mt contact sites are more responsible for the transport of glycerophospholipids, including phosphatidylcholine, phosphatidylethanolamine, phosphatidylinositol and phosphatidylserine (Figure 2C,D). It is questionable whether the transport of various lipids between ER and Mt is transporter‐specific, regulator‐specific, metabolism‐specific and pathology‐specific. The fragments of extended‐synaptotagmin 2 on ER‐Mt contact domains transport some phospholipids, rather than lysoPC, sphingosine or sphingosine‐1‐phosphate, sphingomyelin, phosphoinositides or ceramide.^29^, ^30^ The autophagy‐related proteins regulate the recruitment of the phosphatidylinositol 3‐phosphate kinase complex, lipidation of auto‐related proteins, structure of ER‐Mt contact sites and reconstitution of autophagosome nucleation at ER.^31^

Many transporters contribute to the process of ER‐Mt communication responsible for cholesterol transport. Of those, the cholesterol binding protein Aster‐B regulates cholesterol transport from ER to Mt, fatty acids transport from hydrolysis of cholesterol esters and Mt cholesterol uptake through the putative Mt targeting sequence at the N‐terminus of Aster‐B and activation of ADP‐ribosylation factor1 GTPase.^32^ The interaction of ER with endocytic organelle membrane contact sites during ER‐Mt communication regulates cholesterol transport through endosomal sterol‐binding protein STARD3‐dependent signals of niemann‐Pick type C protein 1 and/or Astra‐B. Effects, and specificities of those regulators were evidenced by target gene deletions with Clustered Regularly Interspaced Short Palindromic Repeats.^33^ One of challenges is to define and confirm the correlation between target gene and protein expressions, between gene expressions and protein activities, and between protein expressions and activities, especially for ER‐associated enzymes. The accumulation of phospholipids and cholesterol resulted from the dysfunction of ER‐Mt communication as second messengers triggers multiple signaling cascades, induces the transcription of drug efflux transporter genes and forms molecular phenomes of cell sensitivity to drugs through alterations of metabolic reprogramming.^34^ Thus, the ER‐Mt communication can change cell sensitivity to external agents and increase cell resistance against drugs.^35^

## 5 ER‐MT COMMUNICATION WITH CA^2+^ TRANSPORT AND PROTEIN SYNTHESIS

**FIGURE 3:**
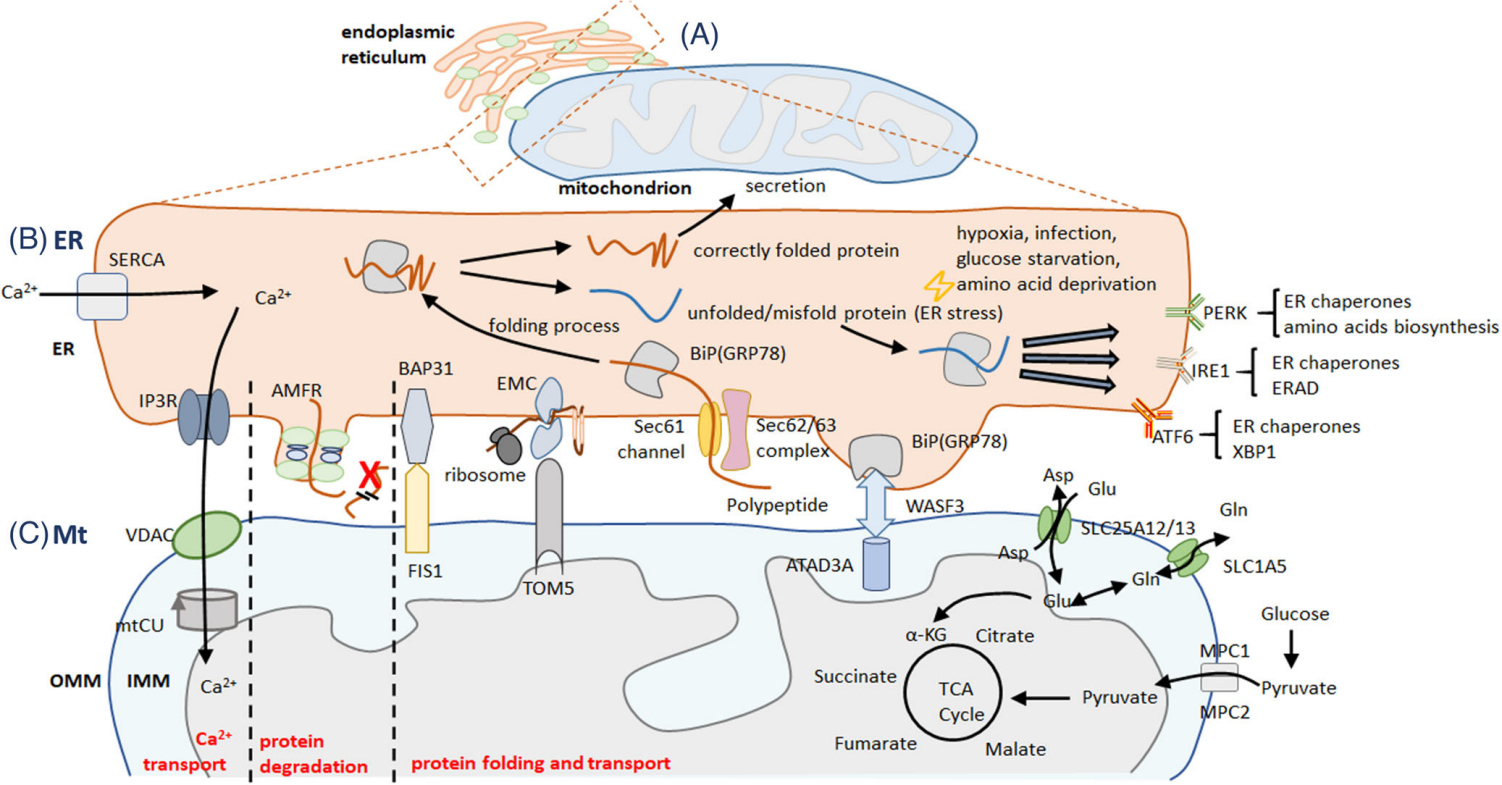
ER‐ Mt regulation of Ca^2+^ transport (A), amino acids and protein metabolism. ER‐Mt communication sites contain several components to impact Ca^2+^ transport including the membrane protein complex IP3R‐VDAC (B), protein degradation, proper protein folding and protein transport. Amino acids are the vital components of polypeptide and can be used to the Mt electron transport chain (C). Abbreviations: Glu, glutamic acid; Gln, glutamine; α‐KG, alpha‐ketoglutarate; TCA cycle, tricarboxylic acid cycle; MPC1, mitochondrial pyruvate carrier 1; MPC2, mitochondrial pyruvate carrier 2; SLC25A12, solute carrier family 25 member 12; SLC25A13, solute carrier family 25 member 13; SLC1A5, solute carrier family 1 member 5; ER, endoplasmic reticulum; Mt, mitochondria; AMFR, autocrine motility factor receptor; GRP78, the 78 kDa glucose‐regulated protein; WASF3, WASP family member 3; ATAD3A, ATPase family AAA domain containing 3A; BAP31, B cell receptor‐associated protein 31; FIS1, fission, mitochondrial 1; EMC, ER membrane protein complex; TOM5, translocase of outer mitochondrial membrane 5; BiP, binding immunoglobulin protein; Sec61, SEC61 translocon; Sec62, SEC62 homolog, preprotein translocation factor; Sec63, SEC63 homolog, protein translocation regulator; OMM, outer mitochondrial membranes; IMM, inner mitochondrial membranes; SERCA, sarco‐endoplasmic reticulum Ca^2+^‐ATPase; ERAD, endoplasmic reticulum‐associated degradation; XBP1, X‐box binding protein 1; IP3R, inositol 1,4,5‐triphosphate (IP3) receptor; VDAC, voltage‐dependent anion channel; mtCU, mitochondrial calcium uniporter; PERK, protein kinase R‐like ER kinase; IRE1, inositol‐requiring enzyme 1; ATF6, activating transcription factor 6

The ER‐Mt communication plays important roles in the signal propagation and transfer of Ca^2+^ and in the control of intracellular Ca^2+^ concentration,^20^ as explained in Figure 3. On the other hand, the Ca^2+^ is one of important regulators in ER‐Mt communication (Figure 3A) from transferring and shuttling to Mt ATP synthesis by activating the tricarboxylic acid cycle. Ca^2+^ induces mitophagy by opening the Mt permeability transition pore and maintains proper ER‐Mt location and distribution by regulating MAM activities.^36^ The membrane protein complex IP3R‐VDAC‐GRP75 is the main transfer pathway between ER and Mt, transferring Ca^2+^ from ER to Mt membrane space.^37^ Through the Mt calcium uniporter in the Mt inner membrane, Ca^2+^ is imported from Mt membrane space into Mt matrix.^38^ MFN2, as a direct ER‐Mt binding protein, also regulates the ER‐Mt interaction and Ca^2+^ transfer, evidenced by the finding that MFN2 deficiency decreased Mt Ca^2+^ uptake.^39^ The Ca^2+^ is transported into ER for loading by the sarco/ER Ca^2+^ ATPase and then released from ER by the transmembrane protein IP3R, and interacts with Mt through the specialized microdomains of MAMs,^40^ as shown in Figure 3B. Of those contacts, the ER protein sigma‐1 receptor, glucose‐regulated proteins, Ca^2+^ uniporters and VDAC are involved in Ca^2+^ transport between ER and Mt by binding with Ca^2+^ or forming a complex with multiple factors.^41^ In addition, ER can sense alterations of reactive oxygen species (ROS), cytosolic adaptor proteins and unfolded proteins with RNA‐dependent protein kinase‐like ER kinase in request of Ca^2+^ presence.^42^ The functional diversity of ER‐Mt contact sites causes the heterogeneity of Ca^2+^ transport and uptake between ER and Mt. It is hardly to monitor structure, function and dynamics of multiple and various linker‐forming proteins and contact nanoscale sites/domains, simultaneously. The accumulations of Ca^2+^ and unfolded proteins and alterations of ER‐associated degradation pathways initiate the development of reticulophagy, during which accumulated unfolded proteins are detected by eukaryotic translation initiation factor 2 alpha kinase 3, ER to nucleus signaling 1 and activating transcription factor 6.^43^ In the physiological conditions, the insulin signaling is activated by increased levels of cytosol Ca^2+^, which is buffered by interacting between Mt and ER through the MAM structure.^44^ While, under cellular stress conditions, the accumulation of Ca^2+^ and ROS, the opening of the Mt permeability transition pore and activation of inflammation in Mt beyond the limitation lead to Mt dysfunction and cell apoptosis.^38^ The disorder of Ca^2+^ transport and regulation consequently causes amino acid metabolism and protein degradation (Figure 3C). Such MAM and Mt dysfunction may furthermore dysregulate Ca^2+^ homeostasis, resulting in insulin insensitivity and the pathogenesis of type 2 diabetes.^45^

## 6 ER‐MT COMMUNICATION WITH AUTOPHAGY AND APOPTOSIS

Many proteins on MAMs directly activate ER stress, ROS over‐production and the process of cell death (e.g., autophagy, mitophagy and ferroptosis), to contribute to pathological responses or restore homeostasis, or both.^46^ The Ca^2+^ is released from ER through the opening of the mitochondrial transition pore to the Mt and then causes apoptosis.^47^ Autophagy is considered as an important process of cell responses to toxins, drugs, antigens and pathogens in the development of tissue inflammation, injury and dysfunction.^48^, ^49^, ^50^ ER‐ and Mt‐dominated reticulophagy and mitophagy are major parts and types of organelle‐specific autophagies.^43^ A number of ER‐ and Mt‐origin genes and proteins contribute to the occurrence of reticulophagy and mitophagy and can be defined as organelle‐, signal‐, regulation‐ and phenome‐specific biomarkers for population screening, early diagnosis and prognostic prediction.^51^, ^52^, ^53^

Apoptosis and autophagy are closely related to Ca^2+^ transporters. IP3R of the IP3R/GRP75/VDAC ER‐Mt tethering complex is one of the important calcium ion channels in ER, to control the release of Ca^2+^ and affect cell metabolism and autophagy.^54^ The Ca^2+^ transport and autophagy are mediated by the PTPIP51‐formed protein complex with VAPB in MAMs.^55^ MFN2 modulates the coupling of ER and Mt, to regulate Mt respiration, autophagy and movement (Figure 1C,D). The over‐expression of MFN2 enhances the ER‐Mt interaction.^56^ The phosphofurin acidic cluster sorting 2 protein maintains the integrity of MAMs, controlling the occurrence of apoptosis and autophagy. The deficiency of those tethering proteins in MAMs fails to form the autophagosomes.^57^ Many proteins on MAM involved in the transfer of Ca^2+^ from ER to Mt are key factors in the occurrence of cell apoptosis.^58^ The deletion of phosphatase and tensin homolog on chromosome 10, a typical tumor suppressor gene on MAM, antagonizes AKT signal, enhances the transfer of Ca^2+^ from ER to Mt and makes cells more sensitive to apoptosis.^59^ The abnormality of ER‐Mt communication reduces the amount of oxidizable fatty acids on the intracellular organelles membrane lipid composition (Figure 2C,D), leading to cell hyposensitivity to oxidative stress and chemotherapy‐induced apoptosis.^60^ Dysfunction of ER‐Mt communication contributes to the pathogenesis of cancer, inflammation, chronic diseases, fibrosis, neurodegeneration, metabolic diseases and drug resistance.^6^, ^38^, ^61^, ^62^, ^63^

## 7 ER‐Mt COMMUNICATION AND POTENTIAL THERAPY TARGETS

**FIGURE 4:**
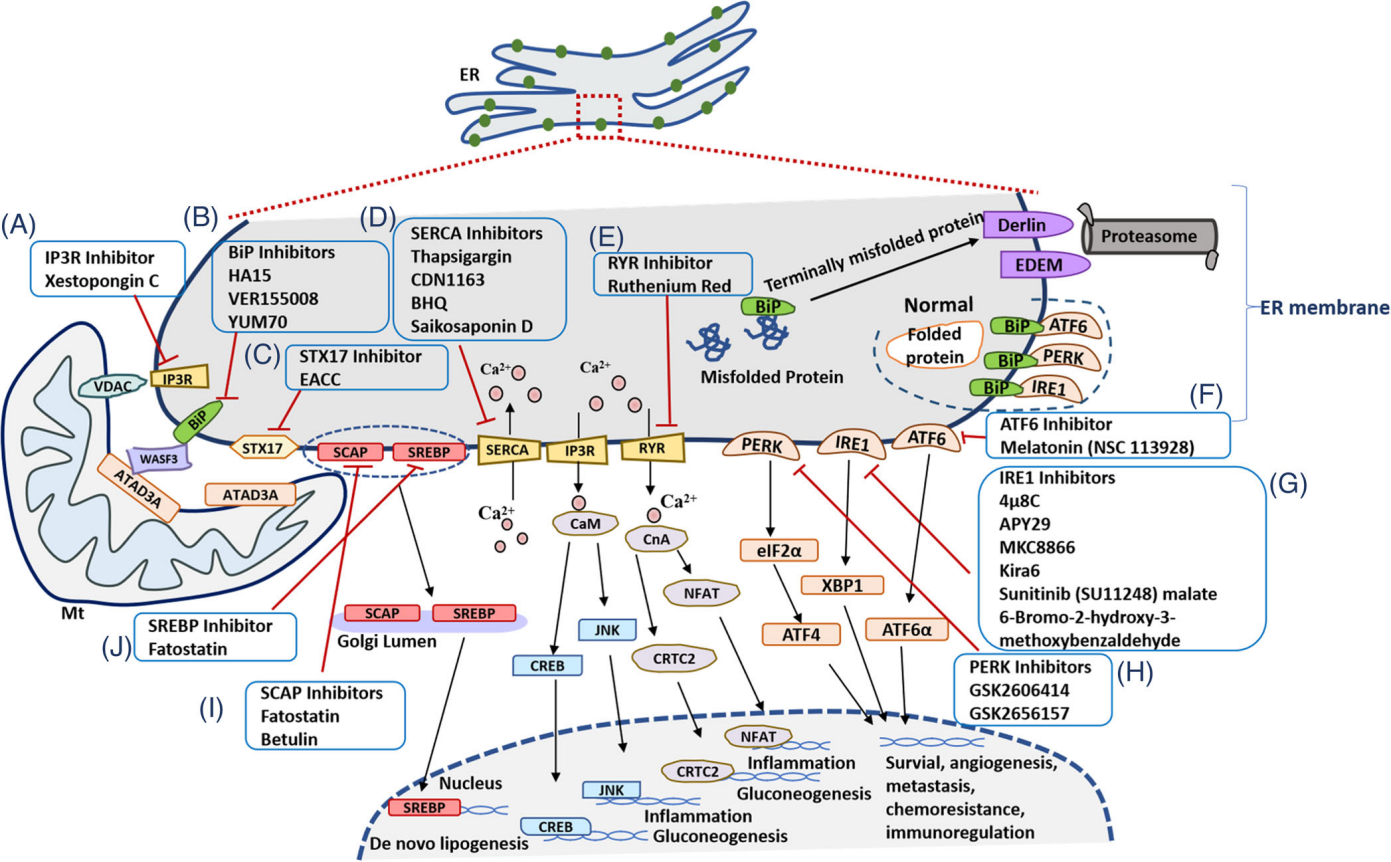
The ER is the endomembrane compartment organelle in the cytoplasm. It serves many major functions in protein synthesis, ER calcium homeostasis and lipid metabolism. IP3R and VDAC regulate Ca^2+^ uptake at mitochondria. Xestopongin C is an inhibitor of IP3R (A). Inhibitors of BiP are HA15, VER155008 and YUM70 (B). STX17 and ATAD3A play a significant role in mitophagy. Inhibitor of STX17 is EACC (C). SERCA, IP3R and RYR play significant roles on maintaining calcium homeostasis. Inhibitors of SERCA are thapsigargin, CDN1163, BHQ and Saikosaponin D (D). Ruthenium red is an inhibitor of RYR (E). The accumulation of unfolded proteins triggers ER stress in its lumen by activating the unfolded protein response (UPR). PERK, IRE1, ATF6 and BiP are involved in this process. Unfolded proteins are eliminated through ER‐associated degradation (ERAD). Melatonin (NSC 113928) inhibits ATF6 (F). IRE1 inhibitors are 4μ8C, APY29, MKC8866, Kira6, Sunitinib (SU11248) malate and 6‐Bromo‐2‐hydroxy‐3‐methoxybenzaldehyde (G). WASF3, providing a bridge between the ER and mitochondria, combine with ATAD3A and BiP. Inhibitors of PERK are GSK2606414 and GSK2656157 (H). The sterol regulatory element‐binding protein family of cholesterol sensors is contained in ER to ensure cholesterol homeostasis. Fatostatin inhibits SREBP and SCAP (J). Betulin also inhibits SCAP (I). Abbreviations: PERK, PKR‐like ER kinase; IRE1, inositol‐requiring enzyme 1; ATF6, activating transcription factor 6; BiP, binding immunoglobulin protein; SREBP, sterol regulatory element‐binding protein; SCAP, SREBP cleavage‐activating protein; SERCA, sarco‐endoplasmic reticulum Ca^2+^‐ATPases; IP3R, inositol‐1,4,5‐triphosphate [IP3] receptors; RYR, ryanodine receptors; CaM, calmodulin; CnA, calcineurin; CRTC2, CREB‐regulated transcription coactivator 2; NFAT, nuclear factor of activated T cells; JNK, c‐Jun N‐terminal kinase; CREB, cAMP‐response element binding protein; eIF2α, eukaryotic translation initiation factor 2α; ATF4, activating transcription factor 4; XBP1, X‐box binding protein 1; EDEM, ER degradation enhancing α‐mannosidase‐like protein; VDAC, voltage‐dependent anion channel 1; WASF, Wiskott–Aldridge syndrome family; STX17, syntaxin‐17; ATAD3A, ATPase family AAA domain containing 3A

The process of ER‐Mt communication can be an important source to generate therapeutic targets for diseases. The inhibitors and modulators of the ER‐Mt communication can be categorized and summarized in Figure 4, according to therapeutic targets. We overview and present inhibitors of IP3R (Figure 4A), BiP (Figure 4B), STX17 (Figure 4C), SERCA (Figure 4D), RYR (Figure 4E), ATF6 (Figure 4F), IRE1 (Figure 4G), PKR‐like ER kinase (PERK) (Figure 4H), SCAP (Figure 4I) and SREBP (Figure 4J). For example, the PERK‐specific inhibitors have the neuroprotective effect on the PERK signaling pathway of the ER‐Mt interface in neurodegeneration.^64^ The silencing of PERK expression restores the impairment of the memory in experimental Alzheimer's disease.^65^ The agonist of ER and Mt link protein Sig‐1R has therapeutic effects on the experimental disease of amyloid lateral sclerosis.^66^ Magalhães Rebelo et al^67^ highlighted small molecular modulators targeting MAMs and divided the potential MAM contact sites that can be used as the drug treatment strategy into three categories: (1) the direct interaction of proteins, (2) inductive effects in the expression of resident proteins and (3) targeted effects on the signal transduction cascade, which ultimately leads to changes in the structure or function of MAM contact sites. Up‐ or down‐expression of those targets directly affects the tightening or loosening of the ER‐Mt contact distance.^68^ The VAPB‐PTPIP51 complex participates in the exchange of Ca^2+^ between ER and Mt and has an effect on the process of autophagy.^55^ In addition, the IP3R‐GRP75‐VDAC complex can correct the final MAM contact sites‐dependent Ca^2+^ defect by changing the interacting molecules as a therapeutic agent.^69^ The transcription factors promote the synthesis of RNA in MAM contact sites. The transcriptional alkaloid berberine is a modulator of MAM contact sites and decreases MFN2 levels, complex I activity and mitochondrial membrane potential experimentally.^70^

Extra‐ER molecules from other organelles participating in the process of ER‐Mt communication are also strong candidates of therapeutic targets. The interaction of mitoguardin in Mt with vesicle‐associated membrane protein‐associated protein of 33 kDa in ER influences the formation of MAMs tensions through phosphorylation of ‘two phenylalanines in an acidic tract' (FFAT)‐like motif and upstream of serine/threonine clusters.^71^ Dysfunction of those extra‐ER‐origin factors may lead to the disorder of MAMs tensions, ER acidification, γ‐secretase activity, Notch signaling, cell polarity and growth, responsible for the development of cancer or neurodegeneration.^72^, ^73^ Although the specificity of transporters and regulators during ER‐Mt communication is to be furthermore clarified, it is important to define the enzymatic activity of the target protein, binding to the drug, and drug efficacy in targeting and modulating the structure and function of ER‐Mt contact sites. Many pyrin domain‐containing 3 triggers induce Ca^2+^ mobilization from ER and change Mt Ca^2+^ uptake in obesity and obesity‐related metabolic diseases with insulin resistance. The ER‐Mt juxtaposition of MAM and Mt calcium uniporter is required for nucleotide‐binding and oligomerization domain‐like receptor, for activation of leucine‐rich repeat and pyrin domain‐containing 3 inflammasome, and for metabolism of omega 3 polyunsaturated fatty acids.^74^ Transporter protein, as a membrane protein in the OMM of the central and peripheral nervous systems, contributes to the translocation of cholesterol to Mt and is considered as a biomarker of injury and inflammation, especially in several inflammatory and neurodegenerative diseases.^75^

Rapid development of biotechnologies provides new insights for understanding molecular mechanisms of the ER‐Mt communication. For example, the systemic lipidome atlas of patients with various subtypes of lung cancer and infection‐ or non‐infection‐induced acute lung injuries were explored using clinical lipidomics and trans‐omics by integrating genomic, lipidomic and clinical phenomes.^76^, ^77^, ^78^, ^79^, ^80^ The integration between systemic lipidomes and leukocyte transcriptomes provides a potential to understand the association between transcriptomic profiles targeting ER‐Mt communication with lipidomic metabolites. Spatial transcriptomics can visually and quantitively image and analyse transcriptomic profiles on tissue surface to provide details of positioning and expression of ER‐Mt communication genes. The integration of single cell RNA sequencing and spatial transcriptomics can reconstruct organelle visualization and the neighbor structure and identify roles of inter‐organelles during cell‐cell interactions in tissues. The question is how to monitor dynamics of interactions between transporters, between regulators and between transporters and regulators in ER‐Mt communication. Chitwood and Hegde^81^ identified the protein associated with the ER translocon complex as an abundant obligate heterodimer of CCDC47 and Asterix with two ER resident membrane proteins and as an intramembrane chaperone, responsible for the engagement of transmembrane domains within the lipid bilayer and for membrane protein biogenesis. The deep understanding of spatial and temporal relations between ER‐Mt locations, communications and dynamics with intra‐ and extra‐cellular phenomes provides special impacts and new insights for improving of spatiotemporal molecular images and medicine.^82^, ^83^

In conclusion, the ER‐Mt communication includes ER‐Mt structural contacts, molecular interactions, chemical exchanges, signal transmissions and inter‐organelle regulations. It is a decisive process of biosynthesis responsible for lipid, amino acid and glucose metabolism regulated by enzymes, transporters and regulators. The elements, structures and functions of the ER‐Mt contact vary among species, organs and responses to external environments. The exact heterogeneity of MAM structural characteristics (e.g., number, length and thickness) among various cell types remains unclear. During the communication, biology‐specific, disease‐specific and metabolism‐specific biomarkers and therapeutic targets can be identified and developed. Regulators of ER‐Mt communication can become potential therapeutic targets for diseases, on basis of the molecular mechanism of ER‐Mt communication. There are still a large number of challenges to classify ER‐Mt contacts according to the function, molecular composition or physical properties.^25^ The spatiotemporal intra‐ and extra‐cellular microenvironments where ER communicates with Mt can be a new source to identify disease biomarkers and therapeutic targets. New strategies of clinical therapy will be developed on basis of the dynamics of ER‐Mt communication. Thus, multi‐dimensional characteristics of molecular changes in different cell species or cell types during ER‐Mt communication have the special value in the improvement of disease diagnosis and therapy.

## CONFLICT OF INTEREST

The authors declare that there is no conflict of interest that could be perceived as prejudicing the impartiality of the research reported.
